# Gene‐specific amplicons from metagenomes as an alternative to directed evolution for enzyme screening: a case study using phenylacetaldehyde reductases

**DOI:** 10.1002/2211-5463.12067

**Published:** 2016-05-13

**Authors:** Nobuya Itoh, Miki Kazama, Nami Takeuchi, Kentaro Isotani, Junji Kurokawa

**Affiliations:** ^1^ Biotechnology Research Center and Department of BiotechnologyToyama Prefectural UniversityImizuToyamaJapan

**Keywords:** bioprospecting, enzyme engineering, gene‐specific amplicons, metagenome, phenylacetaldehyde reductase

## Abstract

Screening gene‐specific amplicons from metagenomes (S‐GAM) is a highly promising technique for the isolation of genes encoding enzymes for biochemical and industrial applications. From metagenomes, we isolated phenylacetaldehyde reductase (*par*) genes, which code for an enzyme that catalyzes the production of various Prelog's chiral alcohols. Nearly full‐length *par* genes were amplified by PCR from metagenomic DNA, the products of which were fused with engineered *par* sequences at both terminal regions of the expression vector to ensure proper expression and then used to construct *Escherichia coli* plasmid libraries. Sequence‐ and activity‐based screening of these libraries identified different homologous *par* genes, *Hpar*‐001 to ‐036, which shared more than 97% amino acid sequence identity with PAR. Comparative characterization of these active homologs revealed a wide variety of enzymatic properties including activity, substrate specificity, and thermal stability. Moreover, amino acid substitutions in these genes coincided with those of *Sar268* and *Har1* genes, which were independently engineered by error‐prone PCR to exhibit increased activity in the presence of concentrated 2‐propanol. The comparative data from both approaches suggest that sequence information from homologs isolated from metagenomes is quite useful for enzyme engineering. Furthermore, by examining the GAM‐based sequence dataset derived from soil metagenomes, we easily found amino acid substitutions that increase the thermal stability of PAR/PAR homologs. Thus, GAM‐based approaches can provide not only useful homologous enzymes but also an alternative to directed evolution methodologies.

**Abbreviations**
ADH: alcohol dehydrogenase
HPAR: homologous phenylacetaldehyde reductase
IPTG: isopropyl‐β‐thiogalactopyranoside
LSADH: *Leifsonia* sp. alcohol dehydrogenase
PAR: phenylacetaldehyde reducatse
S‐GAM: screening gene‐specific amplicons from metagenomes

Metagenomics is an emerging and powerful tool for the isolation of genes, the enzyme products of which may have biochemical and industrial applications 1, 2, 3, 4, 5, 6. PCR amplification of truncated genes from metagenomes can facilitate the identification of genes that encode superior enzymes and yield homologous gene sets that could be used for enzyme screening 7 or DNA shuffling 8, 9. Although previous studies based on PCR‐mediated methods that utilize primers designed from inner conserved sequences have been conducted for several enzymes, including lipase 9, cytochrome P‐450 10, 2,5‐diketo‐d‐gluconic acid reductase 11, and others 4, 6, the methods are generally inefficient and often fail to identify complete functional genes because they lack terminal sequences.

Recently, we reported a novel and efficient approach for isolating *Leifsonia* sp. alcohol dehydrogenase (EC 1.1.1.1) (LSADH)‐related genes from metagenomes 12, 13, 14. This process involved PCR amplification of nearly full‐length genes from metagenomes and their subsequent fusion with the terminal region of an *lsadh*‐expressing vector, enabling the isolation of many novel and diverse *adh* genes and *lsadh* homologs. Moreover, we presented the potential use of novel enzymes as biocatalysts for converting ketones to various anti‐Prelog chiral alcohols 15 at high production levels 16. Thus, screening gene‐specific amplicons from metagenomes (S‐GAM) possesses tremendous biotechnological potential for obtaining novel and valuable gene resources from metagenomes.

In this paper, we describe the construction of a metagenomic library of enzyme genes using an S‐GAM approach that focused on the phenylacetaldehyde reductase (EC 1.1.1.1) (*par*) genes. These genes (circa 1.0 kbp) belong to the zinc‐containing medium‐chain alcohol dehydrogenase family 17, 18, 19, which is comprised of genes that code for enzymes able to catalyze the production of various Prelog's chiral alcohols. Thus, PAR homologs have excellent potential for industrial synthesis of chiral alcohols. We have successfully isolated 35 different active PAR homologs (HPARs) from soil metagenomes. During the analysis of these HPARs, we found that amino acid substitutions within them are scattered but located in quite limited sequence positions. Some of these substitutions completely coincided with those of Sar268 and Har1, which were previously engineered from PAR through application of directed evolution molecular techniques 20, 21. By analyzing the properties of HPARs and comparing their amino acid sequences, we inferred the relationships between each HPAR amino acid sequence and its properties. Using these data, we were able to create mutant HARs with increased thermal stability.

## Results and Discussion

### Metagenome preparation, design of primers, and amplification of *par*‐related genes

We prepared metagenomic DNA samples from 105 soil and compost samples using commercially available DNA isolation kits, and these served as templates without further purification. We recovered detectable amounts of metagenomic DNA from 83 of 105 environmental samples. The average weight of DNA thus obtained from 0.5 g (wet) soil samples was 1.8 ± 1.3 μg by using ISOIL for Beads Beating^™^ kit 16 and 3.1 ± 1.0 μg by using UltraClean Soil DNA^™^ kit. There were no observable differences in DNA quality between the two DNA isolation kits with respect to their function as templates for subsequent PCR amplification.

The primer set was designed from the N‐ and C‐terminal region sequences of *par* and related genes (Fig. S1) that were registered in genome databases and shared between 63% and 82% amino acid sequence identity with PAR. Alignment analysis of these alcohol dehydrogenases (ADH) belonging to the zinc‐containing ADH family (each typically around 350 amino acid residues in length) showed that there are some well‐conserved regions among these genes at both terminal regions. Primers were designed from the conserved sequences ‘E(/S,T)PGPGE(/Q)V(/I)LL’ (from E22 to L30 in PAR) in the N‐terminal region and ‘L(/I)R(/S)GRA(/G)VV(/I)V(/L)’ (from L338 to V345 in PAR) in the C‐terminal region. Primers of 22–28 bp were specifically designed from these sequences so as to overlap with the regions for fusion with the expression vector pSar268‐del‐fus (as described in the Materials and methods section).

We used hot‐start and step‐down PCR protocols to avoid nonspecific amplification and to support the sufficient amplification of DNA using DNA polymerases that offer high fidelity and robust performance 16. Consequently, we were able to detect target bands (of around 1.0 kb) from 42 metagenomic DNA samples from among a total of 83 DNA samples isolated from various soil samples from all over Japan. However, we could not amplify the target genes from bark composts fermenting at 60–80 °C, from which diverse LSADH‐related genes were previously identified 16. Thus, the results clearly suggested that successful amplification of the target genes depends on their properties and the origins of metagenomic DNA samples.

### Screening and analyses of *par*‐related genes from the metagenomic library

PCR‐amplified genes were fused at both terminal regions with the engineered *par* gene (*Sar268*), which codes for an enzyme that much more strongly produces chiral alcohols in the presence of 10–20% (v/v) 2‐propanol than the native PAR enzyme 22; these constructs were then fused with pSAR268‐del‐fus, an expression vector for *Sar268* in *E. coli*. This simple technique was used to ensure proper expression and prevent the low‐level expression of the target genes and thus enabled high‐throughput screening of enzyme function. Approximately 10–15% of the clones that tested positive by colony PCR from each metagenomic sample were subjected to sequencing and enzyme activity analyses. The identified genes were given the descriptor *Hpar* (homologous phenylacetaldehyde reductase) because of their similarity to the *par* gene. In total, 59 clones (30%) of the 200 sequenced clones contained HPAR genes and the remaining clones contained either novel enzyme/protein genes encoding uncharacterized membrane proteins (44%), genes encoding unidentified proteins (18%), or genes that failed to sequence (8%). All *Hpar* gene products were active for some of the substrate ketones tested. We used colony PCR for the first detection step in order to capture a diverse range of novel enzyme genes; however, this step can be omitted if the goal is to simply identify target genes. Indeed, approximately 70% of PCR‐positive clones represented false‐positive identifications of *Hpar* as described above. Therefore, we concluded that function‐ or activity‐based screening is suitable for the S‐GAM approach as the first detection step and that sequence‐based screening including colony PCR is somewhat unnecessary.

After eliminating 24 duplicate genes by comparing the amino acid sequences from all 59 HPAR genes, 35 different HPAR genes were isolated from 18 positive bands on an agarose gel out of 42 total samples electrophoresed after PCR amplification. Notably, the amino acid sequences encoded by the *Hpar* genes obtained via this S‐GAM‐based approach hardly varied; they were all homologs of the native PAR that shared 97.5–98.6% amino acid sequence identity with PAR. Moreover, 10 of 25 duplicate genes were identical to *par*. The data strongly suggested that these genes originated from microorganisms belonging to the genus *Rhodococcus* or a related genus found in soil environments. However, such PAR homologs have not previously been identified by genome mining up‐to‐date DNA databases, although several PAR‐related genes can be obtained by this strictly database‐based approach. Noda‐García *et al*. 23 obtained (βα)_8_ isomerase evolutionary intermediate genes (CAM1 and 2) from a diverse metagenomic sample by mining metagenomic databases (http://data.imicrobe.us/), but discrete groups of this enzyme gene were not identified. Thus a metagenomics approach, especially the S‐GAM method, which relies on the designed primers and the specific origins of metagenomes, does not avoid the inherently biased amplification of target genes and does not always identify a diversity of target genes, even though metagenomic approaches usually reveal the rather high diversity represented by nonculturable microbes.

**Figure 1:**
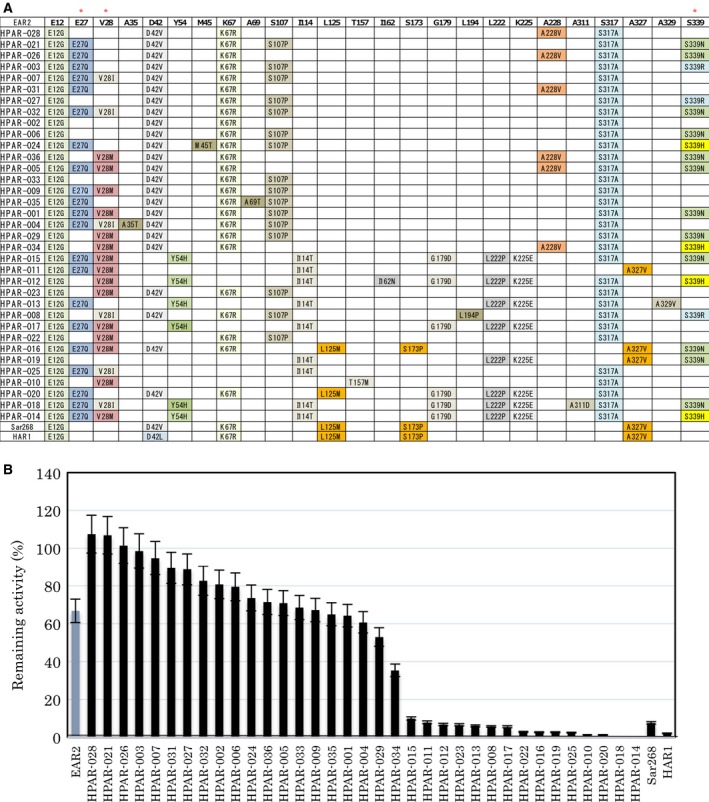
Substitution sites of PAR variants including HPARs (A) and their thermal stabilities (B). Crude enzyme solutions prepared from recombinant *E. coli* cultures were used for each assay. The thermal stability of each enzyme was measured after incubation at 65 °C for 30 min. Bars indicate the SD of three measurements.

Phenylacetaldehyde reductase homologs are tetrameric and chimeric proteins containing Sar268 at both terminal regions, and they each contain 5–10 amino acid substitutions compared with the native PAR subunits: 1 substitution (E12G) was within Sar268 at the N‐terminal region; 1–3 substitutions [E27Q/V28M(I)/S339N(R, H)] were within degenerate primers at the N‐/C‐terminal regions; and 4–7 substitutions were within metagenomic genes. Figure 1A depicts the substitution sites of HPARs relative to those of native PAR, engineered Sar268 20, 22, and Har1 21. We detected major amino acid substitutions of Sar268 and Har1 in the sequences of HPARs–D42V(L), K67R, L125M, S173P, and A327V, each of which is a substitution that substantially increases both the activity of native PAR in the presence of 10–20% (v/v) 2‐propanol and as well as the enzyme's production (as a biocatalyst) of chiral alcohols 20, 22. Moreover, these substitutions coincided not only with the locations but also with the amino acids substituted in Sar268 and Har1, which were engineered by error‐prone PCR and subsequent screening. Even if abundant sequence data for target genes are available in databases, we must generally synthesize the genes and express them in their active forms to clarify the relationship between enzyme sequences and their properties. As described in the next section, we were able to easily determine the enzymatic properties of HPARs. A major advantage of the S‐GAM approach is that it identifies not only their target genes but also their functional information simultaneously.

### Characterization of the thermal stability of HPARs

We measured the differences in thermal stabilities among isolated HPARs because they were easily analyzed in the form of a cell‐free extract without further purification. Figure 1B displays the remaining enzymatic activity after treatment of each HPAR at 65 °C for 30 min, arranged in the descending order of stability. Great differences were observed among HPARs: HPAR‐028, ‐021, ‐026, ‐003, ‐007, ‐031, ‐027, and ‐032 had apparently higher stability than native PAR; HPAR‐015, ‐011, ‐012, ‐023, ‐013, ‐008, ‐017, ‐022, ‐016, ‐019, ‐025, ‐010, and ‐020 had substantially lower stability than PAR (EAR2 is PAR with a His × 6 tag); and HPAR‐018 and ‐014 completely lost their activities. These functional data indicate that amino acid substitutions can increase or decrease the thermal stability of PAR. Substitutions, such as D42V, K67R, S107P, and/or A228V and their combinations, were inferred to increase the thermal stability of the enzyme. On the contrary, Y54H, I114T, G179D, L222P, and/or K225E and their combinations apparently decreased thermal stability (Fig. 1A). The effects of each of these substitutions were evaluated by site‐directed mutagenesis irrespective of their ability to alter the thermal stability of PAR and HPARs.

### Utilization of natural sequence variation to increase the thermal stability of HPAR

**Figure 2:**
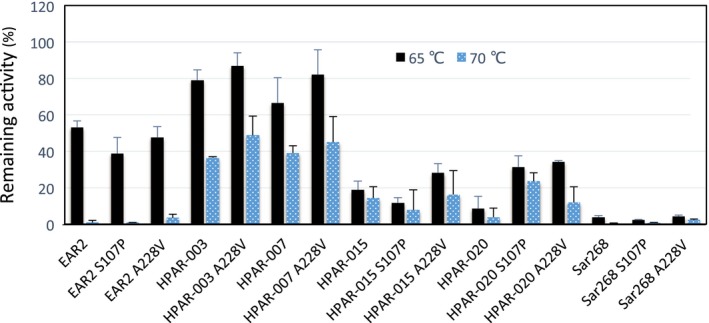
Thermal stability of mutated HPARs. Crude enzyme solutions prepared from recombinant *E. coli* cultures were used for each assay. The thermal stability of each enzyme was measured after incubation at 65 °C or 70 °C for 30 min. Bars indicate the SD of three measurements.

Each S107P and A228V mutation was introduced into PAR (EAR2), HPAR‐003, ‐015, ‐020, and Sar268 (Fig. 2) since all HPARs with high thermal stability originally possessed the D42V and K67R substitutions. Data analysis indicates that substitution S107P does not effectively increase the thermal stabilities of these enzymes, and this substitution has no effect on stability or slightly decreases the stability of EAR2, HPAR‐015, and Sar268, but increases the stability of HPAR‐020. On the other hand, A228V substitutions in HPARs were effective in most contexts, especially in HPAR‐003, ‐015, and ‐020. However, this substitution had no effect in PAR (EAR2) and Sar268. Accordingly, the thermal stabilities of HPAR‐003 (A228V), ‐015 (A228V), and ‐020 (A228V) increased by 10–20% relative to those HPARs without this substitution. Moreover, we inferred that the S107P and A228V mutations in HPAR‐003 and HPAR‐007 increased thermal stability because they originally possessed the S107P mutation. This is reasonable because thermal stability of an enzyme protein depends on the synergistic or accumulative effect of each substitution 24, 25, 26. Our data clearly indicate that natural sequence variants among homologs obtained through the S‐GAM approach, such as S107P and/or A228V, are useful for improving or to altering the enzymatic properties of PAR.

### Other enzymatic differences among HPARs

#### Substrate specificity

**Figure 3:**
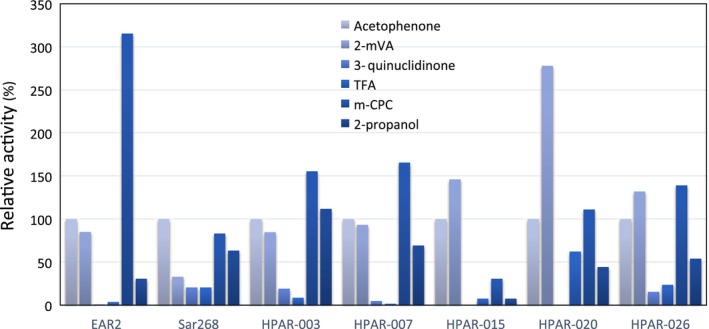
Substrate specificity of HPARs. Purified enzyme solutions prepared from recombinant *E. coli* cultures were used for each assay. All substrate concentrations were 3 mm, except for that of *m*‐CPC, which was at a concentration of 2 mm in 2% DMSO because of its low solubility, and each enzymatic activity for acetophenone was defined as 100%. The activity indicates the mean of two measurements.

As confirmed by the primary screening, all constructed HPARs demonstrated activity in ethyl acetoacetate, although their activities varied. Therefore, the enzymatic activities of representative HPARs (such as HPAR‐003, 007, ‐015, ‐020, and ‐026) toward some ketones, including acetophenone, 2‐methylvaleraldehyde, 3‐quinuclidinone, 2,2,2‐tirfluoroacetophenone, and 2,3′‐dichloroacetophenone (*m*‐CPC), as well as the oxidation of 2‐propanol were evaluated. HPARs were easily purified using Ni‐Sepharose magnetic beads because they contain a His × 6 tag at the C‐terminal region. Specific activity of purified PAR and HPARs toward acetophenone greatly varied, as follows: 1.43 U·mg^−1^ protein for PAR(EAR2), 0.1 U·mg^−1^ for Sar268, 0.08 U·mg^−1^ for HPAR‐003, 0.20 U·mg^−1^ for HPAR‐007, 0.01 U·mg^−1^ for HPAR‐015, 0.003 U·mg^−1^ for HPAR‐020, and 0.10 U·mg^−1^ for HPAR‐026. Although Sar268, HPAR‐003, ‐007, and ‐026 acquired enzymatic activity for 3‐quinuclidinone, PAR, HPAR‐015, and ‐020 demonstrated trace or negligible activity. As shown in Fig. 3, the substrate spectrum of PAR (EAR2), Sar268, and HPARs differed substantially from each other. In previous work comparing and characterizing Sar268, Har1, and PAR 22, we found that these enzymes possess quite variable characteristics. This study confirms data from engineered PAR enzymes and suggests that these HPAR substitutions greatly affect substrate binding even though these substitutions are relatively far away from the active site of the enzyme 22, that is, overall conformational changes to the enzyme proteins greatly affect the substrate specificities of PAR and HPARs. We also reported a similar phenomenon in the ADH homologs of *Pseudomonas* and *Burkholderia* that belong to the short‐chain dehydrogenase/reductase family, as isolated from metagenomes, in which we also found varying substrate specificities 7. Similarly, Nealon *et al*. 27 reported that some point mutations greatly change the substrate specificities and the enantiopreferences of several ADHs.

#### Polar organic solvent 1‐propanol tolerance

**Figure 4:**
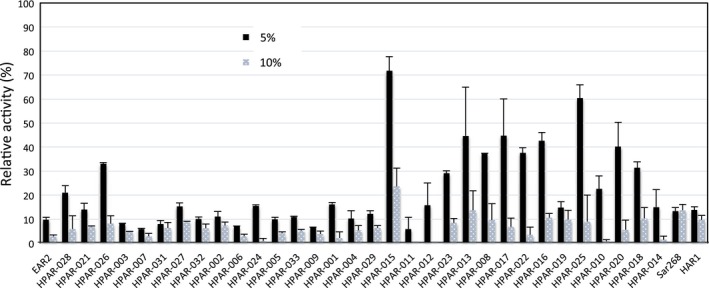
Activity of HPARs in the presence of 5% or 10% (v/v) 1‐propanol. Crude enzyme solutions prepared from recombinant *E. coli* cultures were used for each assay. Bars indicate the SD of three measurements.

The high tolerance of enzymes to organic solvents is a desirable property for enzymes that are intended to be used as biocatalysts for organic synthesis under harsh conditions 28. In the hydrogen‐transfer bioreduction process using PAR or related enzymes, the presence of concentrated 2‐propanol in the reaction mixture is essential to achieve high production levels of chiral alcohols because 2‐propanol serves as a hydrogen donor that regenerates NADH. As such, HPARs were subjected to tolerance testing with 1‐propanol in the reaction mixture because 1‐propanol exhibits the same log*P*_ow_ ratio as 2‐propanol and is a very poor substrate for HPARs. Unfortunately, 2‐propanol was not used for this test because it hinders the enzyme assay conducted with ketone as the substrate. *P*_ow_ is defined as the ratio of the equilibrium concentrations of a dissolved substance in an *n*‐octanol and water two‐phase system 29; the log*P*_ow_ of 1‐/2‐propanol is 0.28. The enzymatic activities of HPARs were measured in 5% and 10% (v/v; 0.65 and 1.3 m) 1‐propanol‐containing reaction mixtures. In general, such polar organic solvents strip surface‐bound water from enzyme proteins and denature their enzyme structures 22, 28. Thus, enzyme activity dramatically decreases as the concentration of 1‐propanol in a reaction mixture increases. As shown in Fig. 4, we observed great differences among HPAR activities under such conditions. HPAR‐028, ‐026, ‐015, ‐023, ‐013, ‐008, ‐017,‐016, ‐019, ‐025, ‐020, and ‐018, as well as engineered enzymes Sar268 and Har1 showed relatively high activity in the presence of 5% or 10% 1‐propanol relative to PAR (EAR2). Furthermore, HPAR‐015, ‐013, ‐016, and ‐025 demonstrated remarkable activity under these conditions. Conversely, most of the thermal‐stable HPARs, such as HPAR‐003 and ‐006 to ‐009, exhibited relatively low activity in the presence of both 5% and 10% 1‐propanol. Among HPARs, we observed an inverse relationship between thermal stability and enzymatic activity in concentrated 1‐propanol. The data suggest that there is a trade‐off between these parameters among HPARs.

## Conclusion

Gene‐specific amplicons from metagenomes is a useful technique for obtaining target genes from metagenomes, as this study has demonstrated with *Hpar* genes. This is the second successful application of the S‐GAM‐based approach followed by its application to LSADH 16. Moreover, just one set of homologs isolated from metagenomes could provide useful information for enzyme engineering because the S‐GAM method can identify not only gene sets but also their enzymatic properties without gene synthesis or subcloning and expression optimization. However, the most important finding in our study was the determination that several amino acid substitutions independently engineered into enzymes by directed evolution were also observed in homologs isolated from naturally occurring metagenomes. This finding suggests that directed evolution, under given selective conditions, can search among possible combinations of amino acid substitutions of homologous enzymes found in nature. Indeed, enzyme homologs in microorganisms arise from the evolutionary processes of (sequential) random mutation and natural selection in the given environments. To artificially obtain such combinations of mutations in enzyme genes, DNA shuffling among genes in a gene family, as reported by Crameri *et al*. 30, is considered to be effective. Our data are compatible with DNA shuffling among genes within a gene family yielding superior enzyme genes. Similarly, ancestral sequence reconstruction 31 and consensus‐based engineering methods 32 have been recognized as effective in increasing the thermal stabilities of enzymes or intrabodies. However, these methods rely upon extensive sequence data already stored in databases. The S‐GAM approach can also be applied to enzyme genes with sparse or unavailable homologous sequence data in databases, like *par* gene sequence data. Moreover, we were able to observe a clear trade‐off in HPARs between thermal stability and enzymatic activity in the presence of a polar organic solvent. Thus, the set of homologs with high similarity obtained via the S‐GAM approach can provide useful and broad information for enzyme engineering.

## Materials and methods

### Metagenome preparation

Metagenomic DNA was extracted from 105 environmental samples including various soils collected from farms, paddy fields, and gardens at independent sites in Japan, and bark composts under fermentation (50–80 °C) in Toyama, Japan using an ISOIL for Beads Beating^™^ kit (Nippon Gene, Tokyo, Japan) or UltraClean Soil DNA^™^ kit (MO BIO Lab. Inc., Carlsbad, CA, USA) according to the manufactures’ protocols. Successful extraction of DNA from the soil and compost samples was confirmed using agarose gel electrophoresis; these DNA samples were used as templates for PCR without further purification.

### Primers, PCR conditions, and cloning of *par*‐related genes

Standard techniques were used for DNA manipulation 33. *E. coli* JM109 cells were used to host *par* genes fused with the pSar268‐del‐fus plasmid. This vector was derived from pSar268 20, which expresses the *Sar268* (*par* mutant) of pUC118, by partial deletion of the *Sar268* gene with *Kpn*I and introduction of a *Not*I site. To introduce fusion sites to both 5′ ends of this plasmid, PCR was performed using KOD FX DNA polymerase (Toyobo, Osaka, Japan) and the following primers: PAR‐091015 sense, 5′‐CGGGCGCGCGGTTGT‐3′ and PAR‐091015 antisense, 5′‐CCTGGCCCGGGCTC‐3′ (the underlined sequences indicate the fusion sites). The reaction mixture contained 10 μL of 2× buffer from the KOD FX kit (Toyobo, Osaka, Japan), 2 nmol of each dNTP, 10 pmol of each primer, circa 50 ng of *Not*I‐treated pSar268‐del plasmid, and 1.0 U of DNA polymerase, yielding a total reaction volume of 20 μL. PCR commenced at 94 °C for 2 min, followed by 25 cycles of 98 °C for 10 s, 63 °C for 30 s, and 68 °C for 4 min. The obtained vector (pSar268‐del‐fus) was linear and contained 15–18 nucleotides at both 5′ ends for fusion with the *hpar* genes amplified directly by PCR using metagenomic DNA samples. DNA sequences were determined for both strands using a capillary DNA sequencer (ABI PRISM 310; Applied Biosystems^®^ Life Technologies, Carlsbad, CA, USA).

In order to obtain gene‐specific amplicons from the metagenomes, PCR was performed with hot‐start and step‐down PCR protocols using KOD Plus Neo, KOD FX Neo DNA polymerase (Toyobo) or Phusion^®^ Hot Start Flex DNA polymerase (New England BioLabs, Tokyo, Japan). Each reaction mixture contained 10 μL of 2× buffer for KOD Plus Neo reactions or 4 μL of 5× Phusion^®^ GC buffer as well as 2 nmol of each dNTP, 10–100 pmol of each primer [GAM‐HPAR‐F 5′‐CGAGCCCGGGCCAGGHSARRTSCTSCTG‐3′ and GAM‐HPAR‐R 5′‐ASSACAACCGCGCGCCCGYKGA‐3′ (again, the underlined sequences indicate the fusion sites)], circa 50 ng of metagenomic DNA, and 0.5 U of DNA polymerase, yielding a total reaction volume of 20 μL. PCR commenced at 94 °C for 2 min, followed by a step‐down protocol: 5 cycles of 98 °C for 10 s and 74 °C for 1 min; 5 cycles of 98 °C for 10 s and 72 °C for 1 min; 5 cycles of 98 °C for 10 s and 70 °C for 1 min; 25 cycles of 98 °C for 10 s and 68 °C for 1 min; and finally the sample was maintained at 68 °C for 7 min.

Amplified fragments were separated by agarose gel electrophoresis, purified using the Wizard^®^ Plus SV Miniprep DNA Purification System (Promega, Madison, WI, USA), and fused between the same sites of pSAR268‐del‐fus with an In‐Fusion^®^ HD Cloning Kit (Clontech, Mountain View, CA, USA). The reaction mixture consisted of circa 100 ng of linearized pSar268‐del‐fus, circa 60 ng of amplified DNA fragments, and 2 μL of In‐Fusion enzyme premix, yielding a total reaction volume of 10 μL, and the mixture was incubated at 50 °C for 15 min. Each plasmid obtained (pHPAR) was electroporated into *E. coli* JM109. The *Hpar* gene of this vector is under the control of the *lac* promoter and is fused to the His × 6‐tag at the *C*‐terminus. Clones were grown at 37 °C on agar plates containing Luria–Bertani (LB) medium [1% (w/v) tryptone, 0.5% (w/v) yeast extract, and 1.0% (w/v) NaCl; pH 7.0] with 100 μg·mL^−1^ ampicillin.

### Screening of *Hpar* genes in *E. coli* clones

Approximately 2000 *E. coli* clones grown on agar plates were screened for *par*‐related genes by colony PCR using GAM‐HPAR‐F and ‐R primers. *E. coli* clones that tested positive after colony PCR were cultured at 37 °C for 24 h with shaking in LB liquid medium (4.0 mL) supplemented with 0.01% ZnCl_2_, 100 μg·mL^−1^ ampicillin, and 0.4 mm isopropyl‐β‐thiogalactopyranoside (IPTG) in test tubes. The cells were collected by centrifugation after cultivation, rinsed, resuspended in 1.0 mL of 50 mm potassium phosphate buffer (KPB; pH 7.0), and disrupted using an ultrasonic oscillator (Ultra Sonic Disrupter UD‐200; Tomy Corp., Tokyo, Japan) for 30 s (three disruption sequences of 10 s followed by a 10‐s interval for cooling). After centrifugation (10 000 ***g*** for 5 min), the supernatant was used as a crude enzyme solution. PAR activity was spectrophotometrically measured at 340 nm and 25 °C in a reaction mixture consisting of 50 mm KPB (pH 7.0), 0.27 μmol of NADH, 3.0 μmol of substrate (ethyl acetoacetate and others), and 10 μL of crude enzyme, yielding a total reaction volume of 1.0 mL 17. The oxidation activity of HPAR was also measured at 340 nm in reaction mixtures with a total volume of 1.0 mL containing 65 μmol of 2‐propanol as the substrate, 1.0 μmol of NAD^+^, 50 mm Tris‐HCl buffer (pH 8.0), and 10 μL of enzyme solution. One unit of enzyme was defined as the amount that converted 1 μmol of NADH (ε = 6220 m ^−1^·cm^−1^) or NAD^+^ in 1 min under these conditions.

### Purification and characterization of recombinant HPARs

Various HPARs, including HAPR‐003, ‐007, ‐015, and ‐020, were purified at 0–4 °C in 20 mm of sodium phosphate buffer containing 20 mm of imidazole (pH 7.4) unless otherwise stated. Washed recombinant *E. coli* cells from each 4‐mL culture broth tubes (cultured at 37 °C for 24 h in a test tube) were suspended in 1.0 mL of buffer and disrupted using an ultrasonic oscillator for 30 s (three disruption sequences of 10 s followed by a 10‐s interval for cooling). After centrifugation (10 000 ***g***, 10 min), the cell‐free extract was subjected to purification. The enzyme was purified using a His Mag Sepharose^™^ Ni bead slurry (200 μL; GE Healthcare Japan, Tokyo, Japan) according to the manufacturer's protocol to obtain high‐purity yields; the cell‐free extract (circa 1.0 mL) of the enzyme was loaded onto the beads equilibrated with the buffer (pH 7.4), incubated for 60 min with mixing for adsorption, washed with the buffer three times, eluted using the buffer containing 500 mm imidazole (pH 7.4), and desalted. The enzyme solution obtained was the purified preparation used to determine the substrate specificity of HPARs.

To characterize the thermal stability of HPARs and their mutant enzymes, each crude enzyme prepared from *E. coli* cultures was incubated at varying temperatures in 50 mm KPB (pH 7.0) for 30 min, and the remaining activity was measured. Moreover, enzymatic activity was measured in the presence of 5% or 10% (v/v) 1‐propanol to evaluate the effect of a polar organic solvent.

### Site‐directed mutagenesis

Site‐directed mutagenesis of *Hpar* was carried out according to the protocols of KOD‐Plus‐Mutagenesis kit (Toyobo) based on the inverse‐PCR technique. The plasmid vectors pHPAR and pSar268, which were purified from *E. coli* recombinant cells using the Wizard^®^ Plus SV Miniprep DNA Purification System (Promega), were used as mentioned previously (in the section ‘Primers, PCR conditions, and cloning of *par*‐related genes’). Mutations were introduced into the *ear2* (*par* tagged with a His × 6), *Hpar*‐003, ‐007, ‐015, ‐020, and *Sar268* genes by using the following primer sets: PAR‐S107P‐Fw, 5′‐CCTCGCGCCCAAGAACTCGGAATCA‐3′ and PAR‐S107P‐Rv, 5′‐GCAATAGTTCTCGAGTCCTTGTGAG‐3′; PAR‐A228V‐Fw, 5′‐TCGAGAACGTCCGCAAGATCACTGG‐3′ and PAR‐A228V‐Rv, 5′‐CCGCGTCCTTGTCGGACAGAAC‐3′. The mutation sites were confirmed by Sanger DNA sequencing of both strands.

### Nucleotide sequence accession numbers

The nucleotide sequences of the identified metagenomic *Hpar*s, *Hpar*‐001 to ‐036 (except *Hpar‐030*, which is missing), were submitted to the DNA Data Bank of Japan under the accession numbers DDBJ: LC060628–LC060636, respectively.

## Author contributions

NI planned experiments and wrote the paper; MK, NT, and KI performed experiments and analyzed the data; JK analyzed the data.

## Supporting information


**Fig. S1.** Primer design for S‐GAM method. Primers were designed based on the alignment of PAR (accession number, DDBJ: AB190261) with known putative ADHs: *Rhodococcus triatomae* (UniProt: M2XIT6, amino acid identity, 82%); *Nocardia nova* (UniProt: W5TCA0, 77%); *Gordonia paraffinivorans* (UniProt: M3TV90, 69%); *Kitasatospora satae* (UniProt: E4NAB2, 65%); *Rhodococcus* sp. (UniProt: A0A059MNJ5, 63%) by clustalw software.
