# Supplementary material for: Gene‐specific amplicons from metagenomes as an alternative to directed evolution for enzyme screening: a case study using phenylacetaldehyde reductases
Source: FEBS Open Bio. 2016 May 13;6(6):566–75. doi: 10.1002/2211-5463.12067 (PMC4887972; doi:10.1002/2211-5463.12067)
Supplement: Supplementary file 1 — Fig. S1. Primer design for S‐GAM method. Primers were designed based on the alignment of PAR (accession number, DDBJ: AB190261) with known putative ADHs: Rhodococcus triatomae (UniProt: M2XIT6, amino acid identity, 82%); Nocardia nova (UniProt: W5TCA0, 77%); Gordonia paraffinivorans (UniProt: M3TV90, 69%); Kitasatospora satae (UniProt: E4NAB2, 65%); Rhodococcus sp. (UniProt: A0A059MNJ5, 63%) by clustalw software. [file FEB4-6-566-s001.pdf]

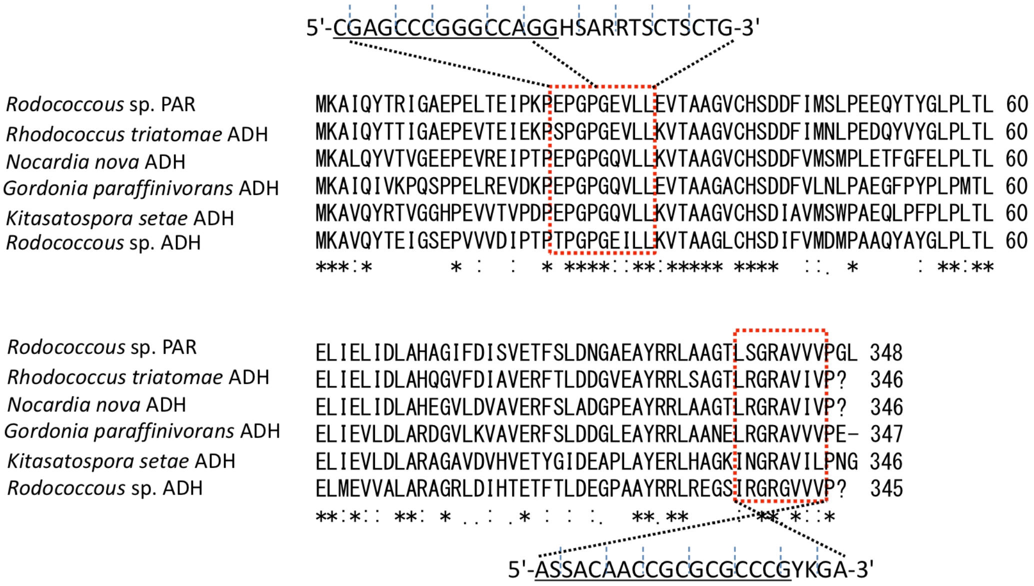

Fig. S1. Primer design for S-GAM method. Primers were designed based on the alignment of PAR (accession number, DDBJ:AB190261) with known putative ADHs: *Rhodococcus triatomae* (UniProt:M2XIT6, amino acid identity, 82%); *Nocardia nova* (UniProt:W5TCA0, 77%); *Gordonia paraffinivorans* (UniProt:M3TV90, 69%); *Kitasatospora satae* (UniProt:E4NAB2, 65%); *Rhodococcus* sp. (UniProt:A0A059MNJ5, 63%) by CLUSTALW software. Identical and similar residues at the terminal regions are shown by asterisks and dots. The conserved amino acid sequences surrounded by a dotted square were used as reference points in the design of primer set. The underlined sequences
